# Pathological and pharmacovigilance monitoring as toxicological imputations of azithromycin and its residues in broilers

**DOI:** 10.14202/vetworld.2024.1271-1280

**Published:** 2024-06-14

**Authors:** Ahmed Fotouh, Doaa Safwat Abdel-Maguid, Maha Abdelhaseib, Rania Samir Zaki, Marwa Darweish

**Affiliations:** 1 Department of Pathology and Clinical Pathology, Faculty of Veterinary Medicine, New Valley University, El Kharga, Egypt; 2 MBA, Marywood University, Pennsylvania, USA; 3 Department of Forensic and Toxicology, Faculty of Veterinary Medicine, New Valley University, El Kharga, Egypt; 4 Department of Food Hygiene, Faculty of Veterinary Medicine, Assiut University, Assiut, Egypt; 5 Department of Food Hygiene, Safety and Technology, Faculty of Veterinary Medicine, New Valley University, El Kharga, Egypt; 6 Department of Pathology, Faculty of Veterinary Medicine, Benha University, 13736, Moshtohor, Toukh, Qaluiobia, Egypt

**Keywords:** antibiotic residues, azithromycin, broiler chicken, liver functions, oxidative stress

## Abstract

**Background and Aim:**

The importance of monitoring antimicrobial residues in food is underlined by increasing worries about food safety and public health. The potential toxicity of azithromycin (Az) on broilers and its impact on chicken meat residues require further investigation. This study assesses Az’s toxicity effects and associated risks in broiler chickens through evaluation.

**Materials and Methods:**

One hundred and twenty chicks were distributed into four equal groups randomly. Each group received different daily oral doses of Az: 200 mg/kg for Az1, 100 mg/kg for Az2, and 50 mg/kg for Az3. The FAz group was given plain water. High-performance liquid chromatography was used to measure Az residue levels in muscle and liver. Oxidative markers (malondialdehyde [MDA], superoxide dismutase [SOD], catalase [CAT]), liver and kidney function tests, and histopathological examination were conducted.

**Results:**

The levels of alanine aminotransferase and aspartate aminotransferase increased in Az1 and Az2 groups from 8 h to 3 days and decreased slightly in Az2 by 7 days, while they remained normal in Az3. The levels of uric acid and creatine in the Az1 and Az2 groups increased from 8 h to 3 days and subsequently decreased in Az2 by the 7^th^ day. Az1 group showed the highest increase in MDA levels within 7 days. With higher Az doses, SOD and CAT levels showed a more significant decrease post-treatment. 9.1 μg/kg Az1 liver had the highest residues, whereas none were detected in muscle.

**Conclusion:**

At higher doses, Az caused significant liver and kidney damage, whereas lower doses had negligible effects. Muscle tissue contains fewer Az residues than liver. Assessing risks and ensuring compliance with regulations necessitate constant surveillance of Az residues in food. The health implications and risk management insights necessitate further investigation into the long-term effects of Az residues.

## Introduction

Approximately one-third of international meat originates from poultry production [1, 2]. This sector has registered robust expansion over the past 50 years [3]. The poultry industry is predicted to be responsible for around half of the meat production growth in the next 10 years [4]. In intensive poultry production, antimicrobial drugs play a significant role [5]. Effective antimicrobial therapy is essential for both livestock health and economic gain in fighting bacterial illnesses. In poultry production, antimicrobial drugs are used for disease prevention, metaphylaxis, and growth promotion [4]. There have been reports of misuse of antimicrobials in food animals, specifically their unauthorized addition to feed or vaccines. A qualitative assessment is necessary for evaluating antimicrobial use in poultry production [6].

Using sub-inhibitory doses of antimicrobial drugs for non-therapeutic reasons in livestock substantially increases antibiotic resistance, particularly for drugs that are vital for human health [4]. High initial doses or insufficient withdrawal before slaughter might result in antimicrobial drugs lingering in animal tissues [7].

Antimicrobial residues may persist in animal tissues due to high initial doses or antibiotic use in food-producing animals which can lead to antibiotic resistance and antibiotic residues in animal products for human consumption. Antimicrobial residues can lead to the development of drug resistance, hypersensitivity, carcinogenicity, mutagenicity, teratogenicity, bone marrow depression, and intestinal flora disruption [8–10].

Macrolides, including Az, are often used antibiotics to treat a range of bacterial infections [11]. Macrolides having bacteriostatic properties inhibit bacterial growth. Az exhibits *in vitro* bactericidal activity against several intracellular pathogens, including those causing chlamydiosis, toxoplasmosis, borreliosis, crypto, and mycobacteriosis [12, 13]. Az is effective against anerobic, Gram-positive, and Gram-negative bacteria [14]. The antiviral properties of the compound have been demonstrated *in vitro* and *in vivo* against several viruses [15]. The reports indicate that it is effective in treating cutaneous pox in pigeons [14]. According to recent reports, Az displays antiviral efficacy against severe acute respiratory syndrome-related coronavirus in both laboratory tests and clinical studies [16, 17]. Az prevents the virus from entering the cell [15]. The immune response against viruses can be boosted by upregulating the production of type I and III interferons [17]. The uncontrolled application of macrolide antibiotics in poultry farming for growth promotion and disease treatment, in combination with premature bird slaughter, can result in antibiotic residues in their meat.

Consumer awareness of chemical residues in food, particularly meat, has surged, leading to heightened public health concerns regarding food safety regulations [18]. The Codex Alimentarius Commission (2011) limits the use of certain macrolides, such as Az, in chicken meat, liver, kidney, and fat to a maximum level of 100 μg/kg [19]. 2005’s Egyptian Organization for Standardization and Quality (EOSQC) guidelines recommend adherence to the Food and Agriculture Organization and European Union standards when setting maximum residue limits for veterinary drugs in chickens, including macrolides [20].

Recently, many researchers have investigated the physiological and pharmacological effects of antimicrobial drugs on birds, along with the presence of drug residues in poultry food [21]. Az is commonly utilized in veterinary and medical practice [22]. In avian medicine, the use of this substance is not extensively documented. This study aimed to evaluate the effects and toxicity risks of Az and its residues in broiler chickens.

## Materials and Methods

### Ethical approval

The New Valley Research Ethics Committee, Egyptian University of New Valley, approved the methods used in the current investigation (Approval number: NVREC-02-3-1-2024).

### Study period and location

This study was conducted from May 2023 to August 2023 in the Animal House at the Faculty of Veterinary Medicine, New Valley University.

### Chemicals

Azithromycin (Az) drug, with the trade name of Zithromax (500 mg) tablet, produced by Pfizer Company, was used in this study.

### Experimental design

One hundred and twenty Ross 1-day-old broiler chicks were used and raised until the age of 32 days in the Animal Laboratory, Assiut University. Health regulations, immunization guidelines, and poultry management methods were followed as per the guidelines of Cruz *et al*. [23]. The birds had unlimited access to water and food during the experiment. They received the super broiler diet throughout their entire growth period. Each pen measured one square meter and had a straw floor. The temperature evolved from 34°C during the initial week, through a 2-week decline to 31°C, to a final stable state of 26°C. The humidity level was set between 42% and 53%. The lighting regimen was constant at 30 L for 24 L:0 D on day 1, reduced to 23 L:1 D from days 2–7, adjusted in gradual increments to reach 20 L:4 D, and then maintained at each respective regime until 32 D of age. 4 groups, each with 30 broiler chicks. 5 days after reaching 20 days of age, they were given Az through a feeding tube and syringe. The birds were divided randomly into four equal groups. The dosages for groups Az1, Az2, and Az3 were 200 mg, 100 mg, and 50 mg, respectively, while the control group (FAz) received water.

### Blood sampling and collection of organs

Blood samples (5 ml) were collected from the wing vein of each bird before medication, 8 h, 3 days, and 7 days post-administration. Blood samples were centrifuged for 10 min at 1006× *g* to obtain clean residual serum. These tissues – liver, kidney, and muscle – have distinct biological roles. Pectoralis major samples were frozen immediately after slaughter and stored at −80°C until analysis. 10% formalin was used to preserve and store tissue samples of liver and kidney for histopathological analysis after cutting them open.

### Biochemical analysis of the liver function tests

Aspartate aminotransferase (AST) and alanine aminotransferase (ALT) enzyme activities were determined using the method by Orhan *et al*. [24]. Measurements were taken using a spectrophotometer from T80 UV/VIS PG instrument Ltd, UK, following the utilization of a commercial kit from BioMérieux-France.

### Biochemical analysis of the kidney function tests

Creatinine levels were assessed using Sies’ method [25], and uric acid levels were measured based on Fotouh *et al*.’s procedure [26]. A BioMérieux commercial kit and a T80 UV/VIS PG instrument were employed.

### Oxidative and antioxidative status

Serum malondialdehyde (MDA) levels were determined using Satoh’s method [27], catalase (CAT) activities were assessed by Aebi’s method [28], and superoxide dismutase (SOD) levels were measured with Biodiagnostic Co.’s commercial kit [29], Egypt.

### Quantification of Az residues using high-performance liquid chromatography (HPLC)

#### Preparation of the tissue samples

According to Leal’s method [30], samples were prepared. 3 g of tissue was extracted using 15 mL of 95:5 (v/v) acetonitrile-methanol mixture and 300 μL of 0.1 mol/L ethylenediaminetetraacetic acid solution. The mixture was sonicated for 15 min and then centrifuged at 1398× *g* for 5 min. The supernatant was filtered through a 0.45 μm polytetrafluoroethylene membrane and then evaporated. After solid-phase extraction cleanup, the sample was injected into the HPLC system.

#### HPLC conditions

A vial containing 100 mg of ≥98% pure Az dihydrate (synonym: Az, Nawah code: AMAA23439, CAS number: 83905-01-5, empirical formula: C38H72N2O, molecular weight: 748.98 g/mol, PubChem ID: 447043) was dissolved in a 100 mL volumetric flask, which was initially filled with 50 mL of mobile phase. The mobile phase used for HPLC analysis consisted of a mixture of water (solvent A) and acetonitrile (solvent B). The initial composition was 60% water and 40% acetonitrile, with a gradient program that changed the proportion to 20% water and 80% acetonitrile over 30 min. Both solvents were HPLC grade and filtered through a 0.22 μm filter before use.

Through sonication for 5 min, the solution’s concentration was raised to 1.0 mg/mL by adding mobile phase until the mark on the flask was reached. Further dilutions of the primary solution were carried out with the mobile phase to achieve concentrations ranging from 50 μg/mL to 100 μg/mL to 200 mg/mL.

HPLC analyses were performed using a Varian, Inc. (USA), quaternary gradient chromatography system, specifically the Varian 920-LC model, which was linked to a photodiode array detector. Varian Galaxie™ Chromatography Software (https://www.chromforum.org/viewtopic.php?t=96654) was utilized for data collection.

The column specifications are validated with a diameter of 2.1 mm × 100 mm and a particle size of 2.6 μm, operated at a temperature of 35°C. The method uses an injection volume of 50 μL, a flow rate of 1 mL/min, and a detection wavelength of 232 nm. The protocol includes a stop time of 15 minutes and a post time of 6 minutes. The mobile phase is composed of 0.05 M phosphoric acid and acetonitrile in a 75:25 ratio (v/v, pH 3.0). The analysis took place at the Multidisciplinary Research Institute of Excellence (no. 8001803) within Cairo’s Faculty of Science.

### Histopathology

The specimens of the liver and kidney were preserved by immersion in 10% neutral-buffered formalin. The tissue samples were processed by dehydrating, embedding in paraffin, and sectioning. Hematoxylin and eosin staining was performed on paraffin-embedded sections, which were cut to a thickness of 5 μm, to visualize the tissue under a light microscope [31].

### Immunohistochemical staining

Paraffin sections from various tissues were stained through immunohistochemistry (IHC) following Hsu *et al*.’s method [32], employing a 5 μg/mL dilution of the mouse monoclonal anti-Caspase-3 Ab (ABM1C12) from Abcam, United Kingdom. Tissue sections in all experimental groups underwent dewaxing and hydration. Using Abcam’s Expose mouse and rabbit-specific HRP/DAB detection kit (ab80436, ready-to-use) (Abcam Company, UK) staining was carried out with the DAB chromogenic agent (ab64238, Abcam Company). Hematoxylin was used for counterstaining. Using a Swift microscope (Leika DM 500) and its Digital leika camera (Wetzlar, Germany), all tissue section images from IHC staining were obtained.

### Statistical analysis

The analysis of variance with the IBM Statistical Package for the Social Science Statistics version 21.0 (IBM Corp., NY, USA) and its least significant difference multiple compare means, and *post hoc* test was utilized to compare the significant differences (p ≤ 0.05) among the four tested means.

## Results

### Assessment of liver function

The mean serum ALT levels in broiler chickens are depicted in Table-1 for the control group (FAz), Az1 (given 200 mg Az), Az2 (given 100 mg Az), and Az3 (given 50 mg Az). During the Az administration, ALT levels did not vary significantly among the groups. 8 h post-medication, the Az1 and Az2 groups had significantly higher ALT levels (44.0 IU/L ± 1.0 and 32.0 IU/L ± 1.3, respectively). The Az3 group’s ALT levels remained consistent with those of the control group. 3 days post-medication, ALT levels remained high in Az1 and Az2 groups compared to Az3 and control groups. By the 7^th^ day, ALT levels in the Az1 group were still elevated at 49.4 ± 3.1 IU/L, while the Az2 and Az3 groups had normal ALT levels akin to the control group.

**Table 1:** Effect of Az on liver function tests of broilers.

Parameters (IU/L) (mean ± SE)	Az1 (200 mg)	Az2 (100 mg)	Az3 (50 mg)	FAz (control)
ALT				
Before	25.2 ± 0.9	25.0 ± 1.3	25.6 ± 1.08	24.4 ± 0.98
8 h	44.0 ± 1.0^a^	32.0 ± 1.3^b^	25.4 ± 1,2^c^	25.2 ± 0.9^c^
3 days	48.8 ± 1.96^a^	34.8 ± 1.5^b^	26.0 ± 1.3^c^	25.0 ± 1.0^c^
7 days	49.4 ± 3.1^a^	25.2 ± 0.9^b^	26.2 ± 1.2^b^	26.0 ± 0.9^b^
AST				
Before	147.4 ± 1.7	146.4 ± 2.0	146.6 ± 1.8	145.8 ± 2.1
8 h	177 ± 3.2^a^	167 ± 3.6^b^	147.4 ± 1.7^c^	146.4 ± 2.0^c^
3 days	181 ± 1.1^a^	173 ± 1.5^b^	149 ± 1.3^c^	147.4 ± 1.7^c^
7 days	168.4 ± 1.9^a^	152.6 ± 1.1^b^	151.2 ± 1.4^bc^	149.2 ± 0.9^c^

Different litters a, b, and c in the same row, indicative at p ≤ 0.05 in relation to the several exposed groups. ALT=Alanine transaminase, AST=Aspartate transaminase, Az=Azithromycin, SE=Standard error

Like AST, the trend was the same. Baseline AST levels ranged from 145.8 IU/L to 147.4 IU/L in all groups. 8 h after treatment, the mean AST levels in all groups had significantly risen compared to the control, with Az1 having a mean of 177 IU/L ± 3.2 and Az2 having a mean of 167 IU/L ± 3.6. Az3 exhibited a comparable mean level of 147.4 ± 1.7 IU/L to the control group. For the next 3 days, Az1 and Az2 showed significantly higher AST levels than the control, with minor variations. On day 7, Az1 and Az2 had lower AST levels compared to Az3.

### Assessment of kidney functions

The mean uric acid levels at various time intervals post-administration are displayed in Table-2. Uric acid levels were equivalent among all groups before treatment. 8 h post-treatment, Az1 had the greatest level (6.8 IU/L ± 0.2), Az2 the next highest (5.9 IU/L ± 0.2), Az3 had a lower level (5.0 IU/L ± 0.2), and the control group had the lowest (4.1 IU/L ± 0.1). After 3 days, the Az1 group exhibited the highest (7.03 IU/L 0.1) uric acid levels, followed by Az2 (6.2 IU/L ± 0.2), Az3 (5.6 IU/L ± 0.2), and the control group (4.0 IU/L ± 0.2). By day 7, Az1 had the highest uric acid level (6.0 IU/L ± 0.2), followed by Az2 (4.3 IU/L ± 0.1), Az3 (4.1 IU/L ± 0.1), and the control group (4.03 IU/L ± 0.1). Az compounds exert a biphasic effect on uric acid levels, with higher doses initially increasing and later decreasing them.

**Table 2:** Effect of Az on kidney function tests of broilers.

Parameters (IU/L) (mean ± SE)	Az1 (200 mg)	Az2 (100 mg)	Az3 (50 mg)	FAz (control)
Uric acid				
Before	4.04 ± 0.1	4.1 ± 0.1	4.04 ± 0.1	4.01 ± 0.1
8 h	6.8 ± 0.2^a^	5.9 ± 0.2^b^	5.0 ± 0.2^c^	4.1 ± 0.1d
3 days	7.03 ± 0.1^a^	6.2 ± 0.2^b^	5.6 ± 0.4^c^	4.0 ± 0.1d
7 days	6.0 ± 0.2^a^	4.3 ± 0.1^b^	4.1 ± 0.1^b^	4.03 ± 0.1^b^
Creatinine				
Before	0.9 ± 0.02	0.9 ± 0.02	0.9 ± 0.02	0.9 ± 0.02
8 h	1.8 ± 0.1^a^	1.3 ± 0.1^b^	1.0 ± 0.1^c^	0.9 ± 0.02^c^
3 days	1.9 ± 0.02^a^	1.7 ± 0.04^b^	1.0 ± 0.01^c^	0.9 ± 0.02^c^
7 days	1.4 ± 0.1^a^	1.3 ± 0.1^b^	1.0 ± 0.01^b^	0.9 ± 0.02^b^

Different letters a, b, and c in the same row, indicative at p ≤ 0.05 in relation to the several exposed groups. Az=Azithromycin, SE=Standard error

### Assessment of the oxidative status biomarkers

The MDA levels, following various time intervals post-administration of distinct Az doses, are detailed in Table-3. Before treatment, all groups had similar MDA levels. At the 8-h mark, Az1 showed the highest MDA level (6.2 [nmol/mL] ± 0.1) compared to Az2 (5.3 [nmol/mL] ± 0.2), Az3 (4.8 [nmol/mL] ± 0.1), and the control group (4.6 [nmol/mL] ± 0.2). At the 3-day mark, Az1 had the highest MDA level (6.3 nmol/mL ± 0.2), compared to Az2 (6.0 nmol/mL ± 0.3), Az3 (6.0 nmol/mL ± 0.3), and the control (at 5.7 nmol/mL ± 0.2). By day 7, MDA levels dropped to 4.8 (nmol/mL) ± 0.2 (Az1), 4.9 (nmol/mL) ± 0.1 (Az2), 4 (nmol/mL) ± 0.2 (Az3), and 4.5 (nmol/mL) ± 0.2 (control group) nmol/mL. The impact of Az compounds on MDA levels varies time-dependently across the study period, as demonstrated by these findings.

**Table 3:** Effect of Az on oxidative status (nmol/mL) of broilers.

Parameters (nmol/ml) (mean ± SE)	Az1 (200 mg)	Az2 (100 mg)	Az3 (50 mg)	FAz (control)
MDA				
Before	4.6 ± 0.2	4.6 ± 0.2	4.7 ± 0.1	4.5 ± 0.2
8 h	6.2 ± 0.1^a^	5.3 ± 0,2^b^	4.8 ± 0.1^c^	4.6 ± 0.2^c^
3 days	6.3 ± 0.2^a^	6.0 ± 0.3^a^	5.7 ± 0.2^a^	4.5 ± 0.2^b^
7 days	4.8 ± 0.2	4.9 ± 0.1	4.7 ± 0.2	4.5 ± 0.2
SOD				
Before	133.2 ± 2.0	133.3 ± 1.7	134.3 ± 1.2	131.5 ± 2.2
8 h	124.3 ± 1.7^b^	128 ± 1.6^b^	133.9 ± 1.8^a^	133.5 ± 1.6^a^
3 days	122.6 ± 2.3^b^	125.1 ± 2.6^b^	132.5 ± 1.9^a^	133.1 ± 2.5^a^
7 days	128.1 ± 2.3	130.2 ± 2.3	132 ± 2.6	132.5 ± 2.4
CAT				
Before	2.8 ± 0.1	2.8 ± 0.1	2.8 ± 0.2	2.8 ± 0.2
8 h	2.2 ± 0.1^b^	2.4 ± 0.1^bc^	2.9 ± 0.1^a^	2.8 ± 0.2^ac^
3 days	2.3 ± 0.2^b^	2.4 ± 0.2^b^	2.9 ± 0.2^a^	2.9 ± 0.1^a^
7 days	2.3 ± 0.3^b^	2.7 ± 0.1^ab^	2.8 ± 0.2^ab^	2.9 ± 0.2^a^

Different letters a, b, and c in the same row, indicative at p ≤ 0.05 in relation to the several exposed groups. MDA=Malondialdehyde, SOD=Superoxide dismutase, CAT=Catalase, Az=Azithromycin, SE=Standard error

The initial SOD activity levels were reported in Table-3, with Az1, AZ2, Az3, and the control group having means of 133.2 ± 2.0, 133.3 ± 1.7, 134.3 ± 1.2, and 131.5 ± 2.2 nmol/mL, respectively. 8 h after treatment, the groups showed significant differences. Az1 had the lowest SOD activity (124.3 [nmol/mL] ± 1.7) compared to Az2 (128.0 [nmol/mL] ± 1.6), Az3 (133.9 [nmol/mL] ± 1.8), and the control group (133.5 [nmol/mL] ± 1.6). At the 3-day mark, SOD activity levels decreased in all treatment groups relative to the control. By the 7^th^ day, SOD activity levels rose slightly to 128.1 ± 2.3 for Az1, 130.2 (nmol/mL) ± 2.3 for Az2, 132.0 (nmol/mL) ± 2.6 for Az3, and 132.5 ± 2.4 for the control group. The study assessed CAT activity levels at various time intervals. After 8 h of administration, CAT activity markedly reduced in groups Az1 and Az2 and remained lowered until day 3. By the 7^th^ day, groups Az1, Az2, and Az3 displayed equal CAT activity levels to the control group.

### Assessment of Az residues

At various time points after dosing, the presence of Az residue in liver samples was assessed, as outlined in Table-4. Eight hours post-administration, the mean residue levels were 104.0 μg/kg ± 4.3 for Az1 and 60.7 μg/kg ± 4.4 for Az2. There were minimal to non-detectable levels in the Az3 and control groups. The mean levels of Az residue in Az1 and Az2 decreased significantly by day 3 to 48.7 μg/kg ± 1.8 and 28.8 ± 2.5, respectively. At day 7, the Az1 and Az2 groups showed mean residue levels of 21.1 μg/kg ± 2.2 and 9.1 μg/kg ± 1.3, respectively, which continued to decline. At this time point, no detectable residues were present in the Az3 and FAz (control) groups.

**Table 4:** Az residues by μg/kg in different broilers tissue.

Sample (mean ± SE)	Az1 (200 mg)	Az2 (100 mg)	Az3 (50 mg)	FAz (control)
Liver				
Before	ND	ND	ND	ND
8 h	104.0 ± 4.3^a^	60.7 ± 4.4^b^	6.2 ± 0.4^c^	ND
3 days	48.7 ± 1.8^a^	28.8 ± 2.5^b^	2.2 ± 0.1^c^	ND
7 days	21.1 ± 2.2^a^	9.1 ± 1.3^b^	ND	ND
Muscle				
Before	ND	ND	ND	ND
8 h	8.7 ± 0.6^a^	4.2 ± 0.2^b^	2.1 ± 0.1^c^	ND
3 days	2.8 ± 0.2^a^	1.8 ± 0.2^b^	1.1 ± 0.4^c^	ND
7 days	0.9 ± 0.04	ND	ND	ND

Different letters a, b, and c in the same row, indicative at p ≤ 0.05 in relation to the several exposed groups. Az=Azithromycin, SE=Standard error

Muscle samples showed varying levels of Az residue at different time points post-administration. The details of Az residue are presented in Table-4. None of the groups showed detectable Az residue levels before administration. By 8 h, significantly higher residue levels were present in both Az1 and Az2 groups, with mean values of 8.7 μg/kg ± 0.6 and 4.2 μg/kg ± 0.2, respectively. By day 3, the mean residue levels in Az1 and Az2 groups declined significantly to 2.8 μg/kg ± 0.2 and 1.8 μg/kg ± 0.2, respectively. The Az1 group had residue levels of 0.9 μg/kg ± 0.04 by day 7, while the Az2 and control groups had non-detectable levels. The decrease in Az residue levels over time in both Az1 and Az2 groups indicates a dose-dependent reduction in muscle tissue. The Az3 and control groups presented minimal to undetectable levels during the study.

### Histopathological findings

**Figure-1:**
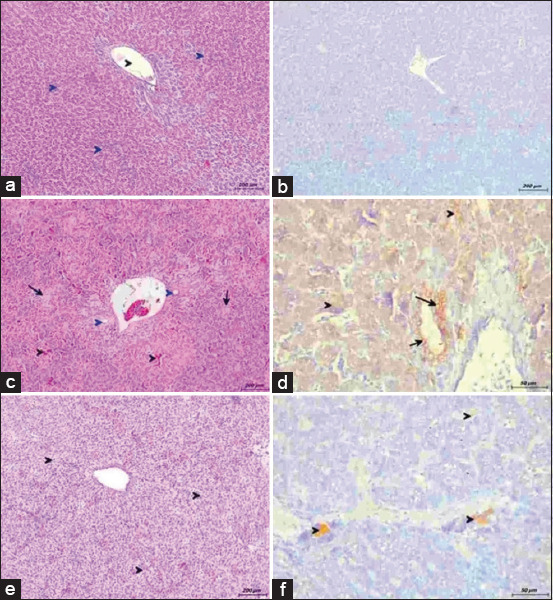
Liver from broilers administered azithromycin (Az) with escalating doses. (a) From control group showing normal hepatocytes (blue arrowhead) radiating around central vein (black arrowhead) (hematoxylin and eosin [H&E], scale bar: 200). (b) No positive reaction for caspases 3 (immunohistochemistry [IHC] stain, scale bar: 200). (c) From group Az 200 mg/kg at 3^rd^-day post-treatment. Congestion and hemorrhage of sinusoids (black arrowhead), focal areas of coagulative necrosis (black arrow), and newly formed blood vessels (blue arrowhead) (H&E, scale bar: 200). (d) Strong positive reaction for caspases-3 in hepatocytes (black arrowhead) and bile ductal epithelium (black arrow) (IHC stain, scale bar: 50). (e) From group Az 200 mg/kg at 7^th^-day post-treatment. Vacuolar degeneration allover hepatic parenchyma (black arrowhead) (H&E, scale bar: 200). (f) Mild positive reaction for caspases-3 in hepatocytes (black arrowhead) (IHC stain, scale bar: 50).

Control liver sections showed normal hepatic parenchyma throughout the experiments. Hepatocytes radiated from central veins, separated by regular sinusoids (Figure-1a). The Az3 group was histopathologically unaltered. At days 1 and 3 post-treatment, the Az2 group showed signs of mild hepatocyte vacuolation, central vein congestion, and sinusoidal dilation. In hepatocytes of the Az1 group, coagulative necrosis focal points are characterized by intense sinusoidal congestion and the emergence of new blood vessels (Figure-1c). Severe vacuolar and fatty degeneration were observed in some cases. The Az1 group was the only treatment that caused vacuolar and fatty degeneration, as evident in Figure-1e.

**Figure-2:**
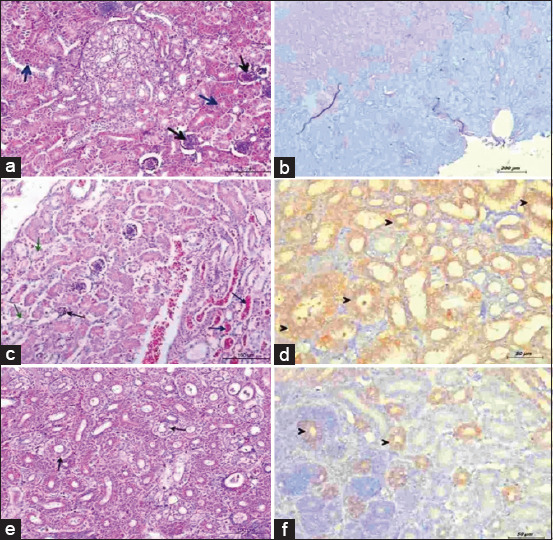
Kidney from broilers administered azithromycin (Az) with escalating doses. (a) The control group showed normal glomeruli (black arrow) and normal convoluted tubules (blue arrow) (hematoxylin and eosin [H&E], scale bar: 200). (b) No positive reaction for caspases-3. (Immunohistochemistry [IHC] stain, scale bar: 200). (c) From group Az 200 mg/kg at 3^rd^-day post-treatment. Atrophied glomerulus (black arrow), vacuolar degeneration of renal lining epithelium (green arrow), and hemorrhage and congestion of blood vessels (blue arrow) (H&E, scale bar: 200). (d) Strong positive reaction for caspases-3 of renal lining epithelium (black arrowhead) (IHC stain, scale bar: 50). (e) From group Az 200 mg/kg at 7^th^-day post-reatment. Mild vacuolar degeneration of some renal tubules (black arrow) (H&E, scale bar: 200). (f) Mild positive reaction for caspases-3 of some renal tubules (black arrowhead) (IHC stain, scale bar: 50).

Each kidney lobe in the control and Az3 groups was made up of several lobules with large cortical tissue encompassing medullary tissue cones (Figure-2a). In the Az2 group, both hemorrhage and congestion were observed. In Az1 group, glomerular atrophy, widened Bowman’s space, vacuolar degeneration of renal tubular epithelium, pyknosis, and karyorrhexis were observed (Figure-2c). Except for vacuolar degeneration of renal tubules, all lesions in the group given the highest dose had diminished by experiment’s end (Figure-2e).

### Immunohistochemical expression of Caspase-3

In Figure-1b, no caspase-3 immunoreactivity was present in the control and Az3 groups’ liver samples. At day 1 and 3 post-treatment, mild caspase-3 expression was observed in hepatocytes surrounding portal triads for the Az2 group. In Figure-1d, strong immunoreactivity of cleaved caspase-3 was detected in hepatocytes and bile ductal epithelium in the Az1 group. All groups, excluding the Az1 group, exhibited no caspase-3 expression by experiment’s end (Figure-1f). The cytoplasm of epithelial cells in both proximal and distal convoluted tubules in the kidneys displayed fine brown granules upon caspase-3 antigen reaction, as shown in Figure-2d. This expression was prevalent in both Az2 and Az1 groups. In the Az3 group, a mild reaction was observed. The control group (Figure-2b) showed no response. In their last sample after medication, the Az2 and Az1 groups had mild Caspase-3 immunoreactivity, as shown in Figure-2f.

## Discussion

Poultry meat production has increased significantly over the past 50 years, now comprising about one-third of global meat production [2]. Due to the requirement for antimicrobial drugs and additives for disease prevention and growth promotion, their excessive usage results in residue accumulation, potentially endangering the food chain [33, 34]. Macrolides, with a broad-spectrum antibacterial action, are extensively utilized in both human and veterinary medicine [35, 36]. Overusing Az in farm animals might result in antibiotic residues in food, posing a contamination risk [15]. Maximum residue limits for inadvertent antibiotic intake in humans are chiefly regulated by food consumption [2, 33–36].

Az is commonly employed to treat lower respiratory tract bacterial infections and boost immunity against bacterial diseases in poultry [37]. At higher dosages of Az, Al-Abdaly *et al*. [38] observed neurotoxic effects on the brains and livers of chick and quail specimens. The available data on Az’s toxicological effects and safe dosage in poultry are inadequate. Az doses higher than usual are associated with increased serum aminotransferase levels, indicating liver dysfunction [39]. Elevated aminotransferases such as ALT and AST indicate liver damage, suggesting a heightened risk of liver toxicity with higher Az doses, consistent with literature on macrolide antibiotics [39]. According to previous studies [40, 41], Az has been reported to cause hepatotoxicity and cardiotoxicity in zebrafish and pathological changes in *Oreochromis niloticus*. According to previous studies [42, 43] tilmicosin macrolides did not modify liver enzyme levels in broiler chickens, macrolide antibiotics’ safety profiles may vary.

Determining the correlation between Az dose and serum aminotransferase levels is essential. Az’s dose-related increase in serum aminotransferase levels necessitates further research into the underlying mechanisms and the duration of potential liver damage. Prolonged administration of Az may heighten the risk of liver damage by boosting the essential proteins and enzymes that support liver function [44]. Lockwood *et al*. [45] reported that Az treatment results in augmented liver function proteins and enzymes due to hepatic membrane damage and leakage. Az’s hepatotoxicity stems from its oxidative properties and interference with normal liver and kidney function, possibly due to the production of harmful free radicals.

Elevated levels of total serum bilirubin and liver enzymes AST and ALT are indicators of liver dysfunction [46]. Global concerns have arisen from reported cases linking Az to acute liver failure [47]. These results are consistent with earlier research suggesting Az-induced hepatotoxicity arises from mitochondrial impairment [47, 48]. More research is required to comprehensively explain how Az causes liver damage through mitochondrial dysfunction and establish safe limits for Az in livestock. A clear comprehension of these mechanisms is essential for informing clinical practice and ensuring Az’s safe use, particularly in patients with pre-existing liver conditions or those at risk of liver complications.

Az induces liver damage by triggering ROS-mediated lipid peroxidation, resulting in hepatocyte membrane destruction and enzyme release [49]. At 8 h and 3 day post-treatment, histopathological findings indicate hepatocyte degeneration, congestion, and sinusoidal dilation. Increased drug doses intensify liver damage [38]. These alterations could be due to direct drug toxicity leading to degeneration and necrosis. Hepatocyte degeneration takes place when homeostasis is disturbed, resulting in the retention of electrolytes and water [50]. Az administration in rats leads to lipid peroxidation in the liver, potentially due to nitric oxide overproduction, inflammation, and cell death [51, 52].

Apoptosis is an energy-dependent process that relies on molecular signaling through various receptors, culminating in caspase activation [50]. In Az2, hepatocytes near portal triads exhibited modest levels of cleaved caspase-3 at 8 h and the 3^rd^ day following treatment. Az1 exhibited significant caspase-3 immunoreactivity in hepatocytes and bile ductal epithelium. Hamza *et al*. [53] reported findings similar to these. Higher Az doses were linked to nephrotoxicity and reduced renal function [54]. High levels of serum creatinine and uric acid suggest potential nephrotoxic effects, hinting at increased risks of renal damage with higher Az doses [54]. Previous study has shown that Az treatment can result in acute interstitial nephritis and renal failure [53]. Az accumulation in the kidney correlates with kidney damage as shown by imaging mass microscopy [55]. Glomerular atrophy, widening of Bowman’s space, and vacuolar degeneration of renal tubular epithelium are the histopathological changes observed in medium to high-dosage groups [54]. Az leads to membrane injury through membrane lipid peroxidation in proximal tubular cells due to high intracellular concentrations [54].

At lower doses, some compounds that cause tubular necrosis induce accelerated apoptosis instead [50]. In both proximal and distal convoluted tubules of the kidneys, Caspase-3 antigen appeared as fine brown granules, mainly in the epithelial cytoplasm. The responses in Az1 and Az2 were strong. These findings align with Ismael Ismael and Elsamman [56], who reported increased caspase-3 activity in Az-treated rats. The mechanisms of Az-induced renal toxicity in poultry might resemble those in humans and other animals. Az can adversely impact kidney function through effects on renal tubular cells, renal hemodynamics, and immune responses. The potential for Az to induce renal toxicity in poultry fosters food safety and public health concerns due to residue risks [57]. Overusing antibiotics contributes to the development of antimicrobial resistance. Effective use of antibiotics and alternative prevention strategies is essential. More research is needed to determine antibiotic guidelines in animal agriculture concerning this issue.

MDAs levels at 8 h and the 3^rd^ day suggest an increase in oxidative stress, possibly due to cytochrome P450-mediated metabolism of drugs or foreign substances in the liver [58]. During Az metabolism, cytochrome P450 produces free radicals and reactive oxygen species (ROS). Mhadhbi *et al*.’s study [59] revealed that protein and lipid damage caused by ROS-induced oxidative stress gives rise to end products such as protein carbonyl and MDA. At higher Az doses, the lower levels of SOD and CAT suggest a possible imbalance in the antioxidant defense system. Az compromises antioxidant defenses, leading to oxidative stress that damages tissue proteins, lipids, and DNA [60]. In previous research [61–63], the Az group con­sistently reported higher MDA levels than the control group.

Residual Az in food animals can pose health risks if consumed excessively or if residues exceed safety limits [64]. Humans can unintentionally consume residues present in animal products. The maximum Az residue concentration was found 8 h post-exposure, with the highest dose group displaying the highest levels, consistent with its extended half-life and significant tissue penetration and accumulation properties [64]. Liver is the primary organ containing Az, followed by the kidney, spleen, lung, and heart [64]. Previous study has also reported similar patterns for other macrolide antibiotics [21].

Amroa *et al*. [21] reported the complete elimination of spiramycin from serum and all tissues except for a slight residual amount after 120 h. The difference in results between the studies may be due to the use of different animal models. In the Hanan region of China, chicken samples contained macrolide antibiotics such as tylosin, Az, and roxithromycin. Two chicken samples had tylosin concentrations of 38.752 and 79.21 μg/kg each, while one sample had Az at a concentration of 27.336 μg/kg. Fohner *et al*. [64] reported below regulatory limit levels of Az in all tissue samples, while no roxithromycin was detected. Oyedeji *et al*. [29] found that Az residues in turkey and chicken meat from Nigeria were below the set limits.

Az may infrequently lead to severe cardiac conditions, including torsades de pointes and polymorphic ventricular tachycardia. In high-risk individuals, a previous study [40] indicated a rise in cardiovascular deaths, while younger adults remain unaffected. Az use can cause hepatotoxicity, characterized by cholestatic jaundice and elevated transaminase levels, within 1–3 weeks. Macrolides’ impact on intestinal motilin receptors leads to gastrointestinal side effects such as nausea and diarrhea. Az treatments may, on rare occasions, induce severe reactions, such as anaphylaxis and Stevens-Johnson syndrome [65,66].

Regulatory agencies established maximum residue limits for Az and other antibiotics in food-producing animals, ensuring their safety in edible tissues. Strict adherence to MRLs and withdrawal periods before slaughter is necessary to ensure the absence of antibiotic residues in food and protect consumer health. Assessing risks and ensuring compliance necessitates monitoring Az residues in food. The long-term health implications of Az residues require further investigation.

## Conclusion

This study is the first to explore the dose-dependent effects of Az on hepatic and renal toxicity in broiler chickens, as well as its residues in liver and muscle tissue. Higher doses of Az correlated with hepatic and renal toxicity, even when lower doses (50 mg) showed minimal changes, similar to the control group. Limiting Az use to lower doses could mitigate toxicity and reduce residue levels in meat and liver. Further research is required to fully understand the toxicological mechanisms of Az-induced hepatotoxicity and residues. Restricting Az dosage to 50 mg or lower can safeguard poultry farming, even though routine monitoring is essential to ensure the absence of Az residues in poultry edible tissues.

## Data availability

The supplementary data can be available from the corresponding author on a request.

## Authors’ Contributions

AF and MD: Design the study, Methodology, and Data analysis. DSA: Methodology, Statistical analysis, and drafted the manuscript. RSZ and MA: Data analysis and reviewed and revised the manuscript. All authors have read, reviewed, and approved the final manuscript.
